# 3-Anilino-5,5-di­methyl­cyclo­hex-2-enone

**DOI:** 10.1107/S160053681400186X

**Published:** 2014-02-05

**Authors:** Houssem Boulebd, Abdelmalek Bouraiou, Sofiane Bouacida, Hocine Merazig, Ali Belfaitah

**Affiliations:** a Laboratoire des Produits Naturels d’Origine Végétale et de Synthèse Organique, PHYSYNOR Université Constantine 1, 25000 Constantine, Algeria; b Unité de Recherche de Chimie de l’Environnement et Moléculaire Structurale, CHEMS, Université Constantine 1, 25000, Algeria; c Département Sciences de la Matière, Faculté des Sciences Exactes et Sciences de la Nature et de la Vie, Université, Oum El Bouaghi, 04000 Oum El Bouaghi, Algeria

## Abstract

In the title mol­ecule, C_14_H_17_NO, the 5,5-di­methyl­cyclo­hex-2-enone moiety is attached to an aniline group, the dihedral angle subtended [54.43 (3)°] indicating a significant twist. The hexaneone ring has a half-chair conformation with the C atom bearing two methyl groups lying 0.6384 (8) Å above the plane of the five remaining atoms (r.m.s. deviation = 0.0107 Å). The crystal packing can be described as alternating layers parallel to (-101), which are consolidated by N—H⋯O hydrogen bonds and C—H⋯π inter­actions.

## Related literature   

For the synthesis of the title compound, see: Amini *et al.* (2013 ▶); Machacek *et al.* (2002 ▶). For its reactivity, see: Wang *et al.* (2007 ▶); Mohammadizadeh *et al.* (2009 ▶); Gao *et al.* (2008 ▶). For our previous work [inspired by Assy (1996 ▶)] on the preparation and the reactivity of imidazole derivatives, see: Zama *et al.* (2013*a* ▶,*b* ▶); Chelghoum *et al.* (2011 ▶); Bahnous *et al.* (2012 ▶) For enamine derivatives as precursors in the synthesis ofcompounds of pharmaceutical inter­est, see: Palko *et al.* (2008 ▶) Park & Jahng (1998 ▶); Tadesse *et al.* (1999 ▶); Thummel & Jahng (1985 ▶); When enamines are treated with alkyl halides, an alkyl­ation occurs to give an iminium salt, see: Adams (2000 ▶); Kempf *et al.* (2003 ▶).
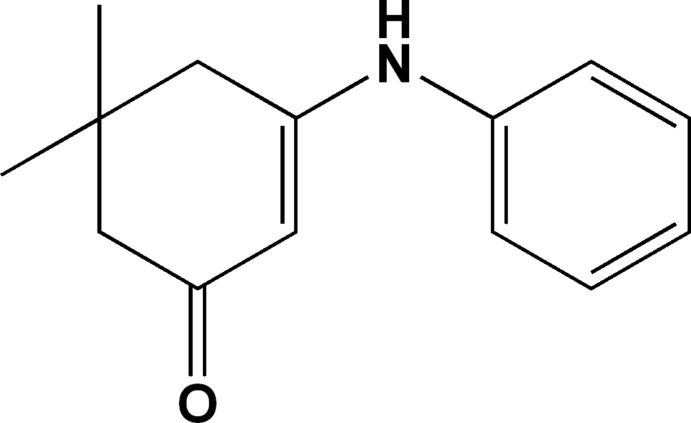


## Experimental   

### 

#### Crystal data  

C_14_H_17_NO
*M* *_r_* = 215.29
Monoclinic, 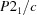

*a* = 10.1766 (19) Å
*b* = 13.159 (2) Å
*c* = 9.2877 (17) Å
β = 104.062 (7)°
*V* = 1206.5 (4) Å^3^
*Z* = 4
Mo *K*α radiation
μ = 0.07 mm^−1^
*T* = 150 K
0.15 × 0.12 × 0.09 mm

#### Data collection  

Bruker APEXII diffractometer
Absorption correction: multi-scan (*SADABS*; Sheldrick, 2002 ▶) *T*_min_ = 0.667, *T*_max_ = 0.747
12403 measured reflections
5021 independent reflections
3873 reflections with *I* > 2σ(*I*)
*R*_int_ = 0.030

#### Refinement  

*R*[*F* ^2^ > 2σ(*F* ^2^)] = 0.047
*wR*(*F* ^2^) = 0.138
*S* = 1.04
5021 reflections
147 parameters
H-atom parameters constrained
Δρ_max_ = 0.45 e Å^−3^
Δρ_min_ = −0.20 e Å^−3^

### 

Data collection: *APEX2* (Bruker, 2001 ▶); cell refinement: *SAINT* (Bruker, 2001 ▶); data reduction: *SAINT*; program(s) used to solve structure: *SIR2002* (Burla *et al.*, 2005 ▶); program(s) used to refine structure: *SHELXL97* (Sheldrick, 2008 ▶); molecular graphics: *ORTEP-3 for Windows* (Farrugia, 2012 ▶) and *DIAMOND* (Brandenburg & Berndt, 2001 ▶); software used to prepare material for publication: *WinGX* (Farrugia, 2012 ▶) and *CRYSCAL* (T. Roisnel, local program).

## Supplementary Material

Crystal structure: contains datablock(s) I. DOI: 10.1107/S160053681400186X/bq2392sup1.cif

Structure factors: contains datablock(s) I. DOI: 10.1107/S160053681400186X/bq2392Isup2.hkl

Supporting information file. DOI: 10.1107/S160053681400186X/bq2392Isup3.cml

CCDC reference:

Additional supporting information:  crystallographic information; 3D view; checkCIF report

## Figures and Tables

**Table 1:** Hydrogen-bond geometry (Å, °) *Cg*1 is the centroid of C10–C15 ring.

*D*—H⋯*A*	*D*—H	H⋯*A*	*D*⋯*A*	*D*—H⋯*A*
N1—H1⋯O1^i^	0.86	2.02	2.8587 (11)	165
C8—H8*A*⋯*Cg*1^ii^	0.96	2.61	3.5547 (12)	157

Symmetry codes: (i) 
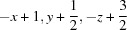
; (ii) 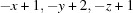.

**Fig. 1.**
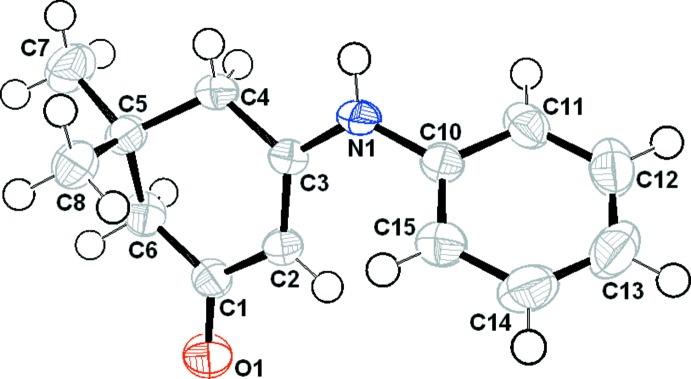
(Farrugia, 2012) The molecular geometry of (I) with the atom-labelling scheme. Displacement ellipsoids are drawn at the 50% probability level. H atoms are represented as small spheres of arbitrary radius.

**Fig. 2.**
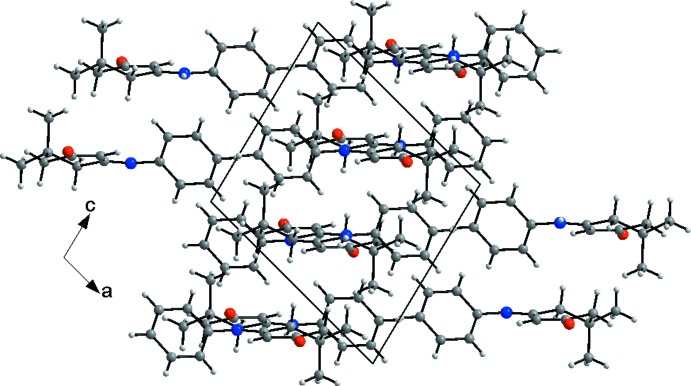
(Brandenburg & Berndt, 2001) Alternating layers parallel to (-101) planes of (I) viewed down the b axis.

**Fig. 3.**
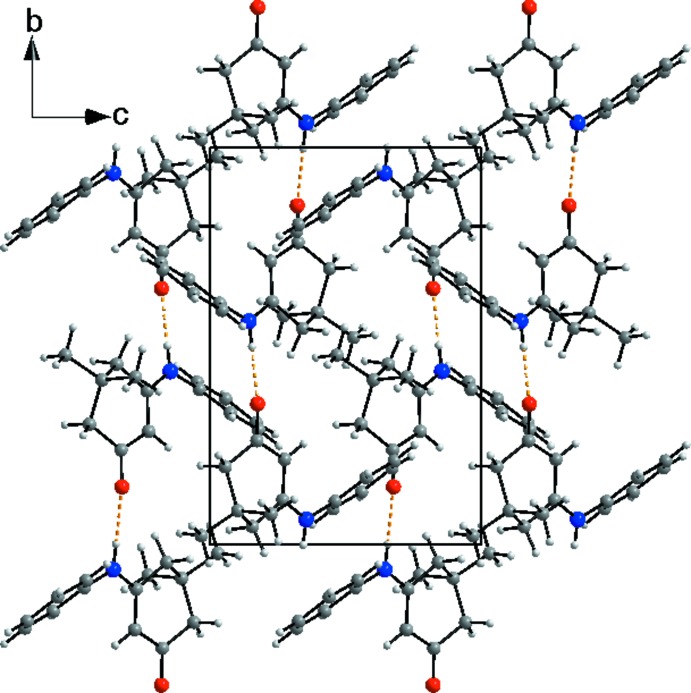
(Brandenburg & Berndt, 2001) A diagram of the layered crystal packing of (I) viewed down the a axis showing hydrogen bond as dashed line.

## supplementary crystallographic information

### 1. Comment

The reaction of primary amines with ketones leads to imines; however, secondary amines give enamines. The preparation of enamines takes place when an aldehyde or ketone containing an α hydrogen is treated with a secondary amine. When enamines are treated with alkyl halides, an alkylation occurs to give an iminium salt *via* an electron transfer from the electron pair on nitrogen, through the C=C to the electrophilic carbon of the alkyl halide (Adams, 2000). In fact, an enamine behaves as a "nitrogen enolate" and generally react as carbon nucleophiles (Kempf *et al.*, 2003). The use of enamine derivatives as intermediates in organic synthesis has been extensively investigated and they proved to be versatile precursors in the synthesis of a large variety of compounds of pharmaceutical interest (Tadesse *et al.*, 1999; Park *et al.*, 1998; Thummel & Jahng 1985; Palko *et al.*, 2008). Inspired by the works of Assy 1996 and also as an extension of our ongoing research on the preparation and the reactivity of imidazole derivatives (Zama *et al.*, 2013*a*; Zama *et al.*, 2013*b*; Chelghoum *et al.*, 2011; Bahnous *et al.*, 2012), we describe herein the single-crystal X-ray structure of the enamine 5,5-dimethyl-3-(phenylamino)cyclohex-2-enone (I). This latter has been recovered from our attempt to coupling the dihydropyridine entity with imidazole unit using a "One Pot reaction" strategy implicating aniline, 5,5-dimethyl-1,3-cyclohexandione and *N*-methylimidazolmethylenemalononitrile. The molecular geometry and the atom-numbering scheme of (I) are shown in Fig. 1. The asymmetric unit of (I) consists of the 5,5-dimethyl-cyclohex-2-enone moiety and its attached phenylamino group. The crystal packing can be described as alternating layers parallel to the (-101)(Fig. 2). It is stabilized by N—H···O hydrogen bond (Fig.3) and C—H···π interactions (Table. 1). These interaction bonds link the molecules within the layers and also link the layers together, reinforcing the cohesion of the structure.

### 2. Experimental

5,5-dimethyl-3-(phenylamino)cyclohex-2-enone (I) has been obtained from the reaction of aniline and 5,5-dimethyl-1,3-cyclohexandione. In a typical reaction, 1 mmol of 5,5-dimethylcyclohex-1,3-dione, and 1 mmol aniline, in ethanol were placed in a 10 ml round-bottomed flask fitted with a condenser and a magnetic stirrer bar. The reaction mixture was stirred at reflux for 24 h, then 1 mmol of *N*-methylimidazolmethylenemalononitrile was added to this solution and the reaction mixture was heated for an additional 24 h. The solvent was distilled off and flash chromatographic purification furnished the 5,5-dimethyl-3-(phenylamino)cyclohex-2-enone I and the recovered *N*-methylimidazolmethylenemalononitrile. Suitable crystals for X-ray experiments of I were obtained by slow evaporation from an ethanol/CH2Cl2 solution at room temperature.

### 3. Refinement

Approximate positions for all the H atoms were first obtained from the difference electron density map. However, the H atoms were situated into idealized positions and the H-atoms have been refined within the riding atom approximation. The applied constraints were as follow: C_aryl_—H_aryl_ = 0.93 Å; C_methylene_—H_methylene_ = 0.97 Å; C_methyl_—H_methyl_ = 0.96 Å and N—H = 0.86 Å; The idealized methyl group was allowed to rotate about the C—C bond during the refinement by application of the command AFIX 137 in *SHELXL97* (Sheldrick, 2008). *U*_iso_(H_methyl_) = 1.5*U*_eq_(C_methyl_) or *U*_iso_(H_aryl_, _methylene_, _amine_) = 1.2 *U*_eq_(C_aryl_, _methylene_ or N).

### Crystal data

**Table:**

C_14_H_17_NO	*F*(000) = 464
*M**_r_* = 215.29	*D*_x_ = 1.185 Mg m^−^^3^
Monoclinic, *P*2_1_/*c*	Mo *K*α radiation, λ = 0.71073 Å
Hall symbol: -P 2ybc	Cell parameters from 4703 reflections
*a* = 10.1766 (19) Å	θ = 2.6–34.3°
*b* = 13.159 (2) Å	µ = 0.07 mm^−^^1^
*c* = 9.2877 (17) Å	*T* = 150 K
β = 104.062 (7)°	Prism, colorless
*V* = 1206.5 (4) Å^3^	0.15 × 0.12 × 0.09 mm
*Z* = 4	

### Data collection

**Table:**

Bruker APEXII diffractometer	3873 reflections with *I* > 2σ(*I*)
Graphite monochromator	*R*_int_ = 0.030
CCD rotation images, thin slices scans	θ_max_ = 34.3°, θ_min_ = 2.6°
Absorption correction: multi-scan (*SADABS*; Sheldrick, 2002)	*h* = −16→14
*T*_min_ = 0.667, *T*_max_ = 0.747	*k* = −13→20
12403 measured reflections	*l* = −12→14
5021 independent reflections	

### Refinement

**Table:**

Refinement on *F*^2^	Primary atom site location: structure-invariant direct methods
Least-squares matrix: full	Secondary atom site location: difference Fourier map
*R*[*F*^2^ > 2σ(*F*^2^)] = 0.047	Hydrogen site location: inferred from neighbouring sites
*wR*(*F*^2^) = 0.138	H-atom parameters constrained
*S* = 1.04	*w* = 1/[σ^2^(*F*_o_^2^) + (0.0725*P*)^2^ + 0.1791*P*] where *P* = (*F*_o_^2^ + 2*F*_c_^2^)/3
5021 reflections	(Δ/σ)_max_ < 0.001
147 parameters	Δρ_max_ = 0.45 e Å^−^^3^
0 restraints	Δρ_min_ = −0.20 e Å^−^^3^

### Special details

**Table:**

Geometry. All e.s.d.'s (except the e.s.d. in the dihedral angle between two l.s. planes) are estimated using the full covariance matrix. The cell e.s.d.'s are taken into account individually in the estimation of e.s.d.'s in distances, angles and torsion angles; correlations between e.s.d.'s in cell parameters are only used when they are defined by crystal symmetry. An approximate (isotropic) treatment of cell e.s.d.'s is used for estimating e.s.d.'s involving l.s. planes.
Refinement. Refinement of *F*^2^ against ALL reflections. The weighted *R*-factor *wR* and goodness of fit *S* are based on *F*^2^, conventional *R*-factors *R* are based on *F*, with *F* set to zero for negative *F*^2^. The threshold expression of *F*^2^ > σ(*F*^2^) is used only for calculating *R*-factors(gt) *etc*. and is not relevant to the choice of reflections for refinement. *R*-factors based on *F*^2^ are statistically about twice as large as those based on *F*, and *R*- factors based on ALL data will be even larger.

### Fractional atomic coordinates and isotropic or equivalent isotropic displacement parameters (Å^2^)

**Table:**

	*x*	*y*	*z*	*U*_iso_*/*U*_eq_	
C1	0.64385 (8)	0.73838 (6)	0.82567 (9)	0.01988 (15)	
C2	0.53143 (8)	0.78529 (6)	0.72449 (9)	0.01973 (15)	
H2	0.4742	0.7455	0.6531	0.024*	
C3	0.50602 (8)	0.88768 (6)	0.73022 (9)	0.01767 (14)	
C4	0.59803 (8)	0.95491 (6)	0.84239 (10)	0.02194 (16)	
H4A	0.5593	0.963	0.9274	0.026*	
H4B	0.6012	1.0216	0.7987	0.026*	
C5	0.74309 (8)	0.91494 (6)	0.89647 (9)	0.01943 (15)	
C6	0.73478 (9)	0.80376 (6)	0.94261 (9)	0.02232 (16)	
H6A	0.8253	0.775	0.9661	0.027*	
H6B	0.7019	0.8017	1.0322	0.027*	
C7	0.81684 (10)	0.97776 (8)	1.03068 (11)	0.0320 (2)	
H7A	0.7703	0.9715	1.1086	0.048*	
H7B	0.8185	1.0478	1.0024	0.048*	
H7C	0.908	0.9533	1.0652	0.048*	
C8	0.81959 (9)	0.92285 (7)	0.77337 (10)	0.02490 (17)	
H8A	0.8268	0.9929	0.7477	0.037*	
H8B	0.7712	0.886	0.6876	0.037*	
H8C	0.9086	0.8945	0.8079	0.037*	
C10	0.29024 (8)	0.89121 (6)	0.53767 (9)	0.01887 (15)	
C11	0.15721 (8)	0.90959 (7)	0.54586 (10)	0.02316 (16)	
H11	0.1403	0.95	0.6215	0.028*	
C12	0.04984 (9)	0.86746 (7)	0.44081 (12)	0.0306 (2)	
H12	−0.0388	0.88	0.4461	0.037*	
C13	0.07470 (11)	0.80686 (7)	0.32830 (13)	0.0351 (2)	
H13	0.003	0.7781	0.2587	0.042*	
C14	0.20729 (11)	0.78931 (8)	0.31997 (12)	0.0343 (2)	
H14	0.2239	0.7488	0.2443	0.041*	
C15	0.31524 (9)	0.83168 (7)	0.42365 (10)	0.02586 (18)	
H15	0.4037	0.8203	0.4168	0.031*	
N1	0.39813 (7)	0.93664 (5)	0.64459 (8)	0.02126 (14)	
H1	0.3945	1.0014	0.6559	0.026*	
O1	0.66676 (7)	0.64544 (5)	0.82303 (9)	0.03086 (17)	

### Atomic displacement parameters (Å^2^)

**Table:**

	*U*^11^	*U*^22^	*U*^33^	*U*^12^	*U*^13^	*U*^23^
C1	0.0190 (3)	0.0142 (3)	0.0280 (4)	−0.0002 (2)	0.0086 (3)	0.0044 (3)
C2	0.0182 (3)	0.0134 (3)	0.0268 (4)	−0.0001 (2)	0.0040 (3)	−0.0003 (3)
C3	0.0170 (3)	0.0144 (3)	0.0217 (3)	0.0010 (2)	0.0048 (3)	−0.0012 (2)
C4	0.0214 (3)	0.0170 (3)	0.0261 (4)	0.0017 (3)	0.0032 (3)	−0.0060 (3)
C5	0.0198 (3)	0.0172 (3)	0.0202 (3)	−0.0011 (3)	0.0027 (3)	−0.0009 (3)
C6	0.0225 (3)	0.0210 (4)	0.0226 (3)	0.0010 (3)	0.0036 (3)	0.0053 (3)
C7	0.0320 (4)	0.0310 (5)	0.0280 (4)	−0.0039 (4)	−0.0024 (4)	−0.0070 (4)
C8	0.0245 (4)	0.0214 (4)	0.0300 (4)	−0.0041 (3)	0.0092 (3)	0.0014 (3)
C10	0.0191 (3)	0.0144 (3)	0.0221 (3)	0.0013 (2)	0.0031 (3)	0.0013 (2)
C11	0.0212 (3)	0.0206 (3)	0.0278 (4)	0.0043 (3)	0.0061 (3)	0.0046 (3)
C12	0.0201 (4)	0.0254 (4)	0.0437 (5)	−0.0011 (3)	0.0025 (3)	0.0103 (4)
C13	0.0317 (5)	0.0228 (4)	0.0420 (5)	−0.0068 (3)	−0.0080 (4)	0.0015 (4)
C14	0.0397 (5)	0.0256 (4)	0.0328 (5)	0.0008 (4)	−0.0004 (4)	−0.0097 (4)
C15	0.0254 (4)	0.0237 (4)	0.0278 (4)	0.0026 (3)	0.0052 (3)	−0.0053 (3)
N1	0.0210 (3)	0.0135 (3)	0.0266 (3)	0.0034 (2)	0.0007 (3)	−0.0026 (2)
O1	0.0290 (3)	0.0135 (3)	0.0487 (4)	0.0018 (2)	0.0067 (3)	0.0065 (3)

### Geometric parameters (Å, º)

**Table:**

C1—O1	1.2463 (10)	C7—H7C	0.96
C1—C2	1.4320 (11)	C8—H8A	0.96
C1—C6	1.5126 (12)	C8—H8B	0.96
C2—C3	1.3754 (11)	C8—H8C	0.96
C2—H2	0.93	C10—C15	1.3895 (12)
C3—N1	1.3523 (10)	C10—C11	1.3954 (12)
C3—C4	1.5069 (11)	C10—N1	1.4209 (10)
C4—C5	1.5323 (12)	C11—C12	1.3911 (13)
C4—H4A	0.97	C11—H11	0.93
C4—H4B	0.97	C12—C13	1.3857 (16)
C5—C7	1.5316 (12)	C12—H12	0.93
C5—C6	1.5326 (12)	C13—C14	1.3895 (16)
C5—C8	1.5351 (12)	C13—H13	0.93
C6—H6A	0.97	C14—C15	1.3896 (13)
C6—H6B	0.97	C14—H14	0.93
C7—H7A	0.96	C15—H15	0.93
C7—H7B	0.96	N1—H1	0.86
			
O1—C1—C2	122.25 (8)	C5—C7—H7C	109.5
O1—C1—C6	119.18 (7)	H7A—C7—H7C	109.5
C2—C1—C6	118.54 (7)	H7B—C7—H7C	109.5
C3—C2—C1	121.65 (7)	C5—C8—H8A	109.5
C3—C2—H2	119.2	C5—C8—H8B	109.5
C1—C2—H2	119.2	H8A—C8—H8B	109.5
N1—C3—C2	125.26 (7)	C5—C8—H8C	109.5
N1—C3—C4	113.97 (7)	H8A—C8—H8C	109.5
C2—C3—C4	120.72 (7)	H8B—C8—H8C	109.5
C3—C4—C5	114.36 (7)	C15—C10—C11	119.93 (8)
C3—C4—H4A	108.7	C15—C10—N1	121.09 (7)
C5—C4—H4A	108.7	C11—C10—N1	118.95 (8)
C3—C4—H4B	108.7	C12—C11—C10	119.99 (9)
C5—C4—H4B	108.7	C12—C11—H11	120
H4A—C4—H4B	107.6	C10—C11—H11	120
C7—C5—C4	108.91 (7)	C13—C12—C11	120.13 (9)
C7—C5—C6	109.70 (7)	C13—C12—H12	119.9
C4—C5—C6	107.77 (7)	C11—C12—H12	119.9
C7—C5—C8	109.43 (7)	C12—C13—C14	119.69 (9)
C4—C5—C8	110.80 (7)	C12—C13—H13	120.2
C6—C5—C8	110.20 (7)	C14—C13—H13	120.2
C1—C6—C5	114.07 (7)	C13—C14—C15	120.64 (10)
C1—C6—H6A	108.7	C13—C14—H14	119.7
C5—C6—H6A	108.7	C15—C14—H14	119.7
C1—C6—H6B	108.7	C10—C15—C14	119.61 (9)
C5—C6—H6B	108.7	C10—C15—H15	120.2
H6A—C6—H6B	107.6	C14—C15—H15	120.2
C5—C7—H7A	109.5	C3—N1—C10	126.13 (7)
C5—C7—H7B	109.5	C3—N1—H1	116.9
H7A—C7—H7B	109.5	C10—N1—H1	116.9
			
O1—C1—C2—C3	178.90 (8)	C8—C5—C6—C1	−69.18 (9)
C6—C1—C2—C3	0.93 (12)	C15—C10—C11—C12	0.66 (12)
C1—C2—C3—N1	−176.05 (8)	N1—C10—C11—C12	178.82 (7)
C1—C2—C3—C4	1.17 (12)	C10—C11—C12—C13	0.27 (13)
N1—C3—C4—C5	−157.43 (7)	C11—C12—C13—C14	−0.70 (15)
C2—C3—C4—C5	25.06 (11)	C12—C13—C14—C15	0.19 (16)
C3—C4—C5—C7	−168.72 (7)	C11—C10—C15—C14	−1.17 (13)
C3—C4—C5—C6	−49.78 (9)	N1—C10—C15—C14	−179.28 (8)
C3—C4—C5—C8	70.86 (9)	C13—C14—C15—C10	0.74 (15)
O1—C1—C6—C5	152.80 (8)	C2—C3—N1—C10	2.19 (14)
C2—C1—C6—C5	−29.16 (11)	C4—C3—N1—C10	−175.20 (7)
C7—C5—C6—C1	170.28 (7)	C15—C10—N1—C3	−54.91 (12)
C4—C5—C6—C1	51.84 (9)	C11—C10—N1—C3	126.96 (9)

### Hydrogen-bond geometry (Å, º)

Cg1 is the centroid of C10–C15 ring.

**Table:**

*D*—H···*A*	*D*—H	H···*A*	*D*···*A*	*D*—H···*A*
N1—H1···O1^i^	0.86	2.02	2.8587 (11)	165
C8—H8*A*···*Cg*1^ii^	0.96	2.61	3.5547 (12)	157

Symmetry codes: (i) −*x*+1, *y*+1/2, −*z*+3/2; (ii) −*x*+1, −*y*+2, −*z*+1.
